# Polyether Single and Double Crystalline Blends and the Effect of Lithium Salt on Their Crystallinity and Ionic Conductivity

**DOI:** 10.3390/polym13132097

**Published:** 2021-06-25

**Authors:** Jorge L. Olmedo-Martínez, Michele Pastorio, Elena Gabirondo, Alessandra Lorenzetti, Haritz Sardon, David Mecerreyes, Alejandro J. Müller

**Affiliations:** 1 POLYMAT and Department of Polymers and Advanced Materials: Physics, Chemistry and Technology, Faculty of Chemistry, University of the Basque Country UPV/EHU, Paseo Manuel de Lardizabal 3, 20018 Donostia-San Sebastián, Spain; jolmedo001@ikasle.ehu.es (J.L.O.-M.); elena.gabirondo@polymat.eu (E.G.); haritz.sardon@ehu.eus (H.S.); david.mecerreyes@ehu.es (D.M.); 2 Department of Industrial Engineering, University of Padova, via Marzolo, 9, 35131 Padova, Italy; pastorio.michele@gmail.com (M.P.); alessandra.lorenzetti@unipd.it (A.L.); 3 IKERBASQUE, Basque Foundation for Science, 48009 Bilbao, Spain

**Keywords:** polyethers, PEO blends, ionic conductivity, isothermal crystallization rate

## Abstract

In this work, blends of Poly(ethylene oxide), PEO, and poly(1,6-hexanediol), PHD, were prepared in a wide composition range. They were examined by Differential Scanning Calorimetry (DSC), Polarized Light Optical Microscopy (PLOM) and Wide Angle X-ray Scattering (WAXS). Based on the results obtained, the blends were partially miscible in the melt and their crystallization was a function of miscibility and composition. Crystallization triggered phase separation. In blends with higher PEO contents both phases were able to crystallize due to the limited miscibility in this composition range. On the other hand, the blends with higher PHD contents display higher miscibility and therefore, only the PHD phase could crystallize in them. A nucleation effect of the PHD phase on the PEO phase was detected, probably caused by a transference of impurities mechanism. Since PEO is widely used as electrolyte in lithium batteries, the PEO/PHD blends were studied with lithium bis(trifluoromethanesulfonyl) imide (LiTFSI), and the effect of Li-salt concentration was studied. We found that the lithium salt preferentially dissolves in the PEO phase without significantly affecting the PHD component. While the Li-salt reduced the spherulite growth rate of the PEO phase within the blends, the overall crystallization rate was enhanced because of the strong nucleating effect of the PHD component. The ionic conductivity was also determined for the blends with Li-salt. At high temperatures (>70 °C), the conductivity is in the order of ~10^−3^ S cm^−1^, and as the temperature decreases, the crystallization of PHD was detected. This improved the self-standing character of the blend films at high temperatures as compared to the one of neat PEO.

## 1. Introduction

Aliphatic polyethers are a broad class of polymers, nowadays used in a wide range of fields [1]. However, their industrial applications are limited to short-chain aliphatic polyethers. Commercially available short chain aliphatic polyethers such as polyethylene oxide (PEO), polypropylene oxide (PPO) or polytetrahydrofuran (PTHF) are industrially obtained by ring-opening polymerization [2,3,4]. Recently, a sustainable route for the synthesis of medium- to long-chain aliphatic polyethers has been reported [5]. This synthetic method open the possibility of tuning the length of the aliphatic chain of polyethers, preparing different copolymers and fine tuning of their *T_m_* and crystallinity [5,6,7].

One of the actual applications of PEO is as solid polymer electrolytes (SPEs) for lithium batteries of electric vehicles [8]. Polyethylene oxide (PEO) stands out as the main host polymer for polymer electrolytes, due to its excellent capability to dissolve lithium salts, and because it exhibits high ionic conductivity values at relative high temperatures (>70 °C) [9,10,11]. In particular, the polyethylene oxide (PEO)/lithium bis(trifluoromethanesulfonyl) imide (LiTFSI) system has been widely studied because of the high dissociation and plasticizing abilities of LiTFSI, that lead to better ionic conductivities compared to other salts [12,13]. However, the high crystallinity and the low mechanical strength of PEO are still issues to be improved [14]. Specifically, the crystallization limits the ionic conductivity, due to the reduction of amorphous conductivity pathways caused by the spherulitic growth [15]. Many strategies have been attempted to improve the comprehensive performance of PEO-based electrolytes, such as: filler addition (e.g., nanoparticles [16,17]), synthesis of PEO copolymers (random, block [18] or graft [19]), crosslinking [20] or blending techniques [14,21,22]. In particular, blending can suppress crystallization, increasing the percentage of the amorphous phase and as a result the ionic conductivities of polymer electrolytes are improved. Compared to copolymerization of PEO, polymer blending technique is convenient, efficient, and low cost. Indeed, the main advantages of polymer electrolytes prepared via blending method are the simplicity of preparation and easy control of physical properties by compositional change, overcoming the serious drawback of preparing electrolytes by nontrivial synthesis methods, which are not very suitable for practical applications.

For this reason, different polymer blends have been studied to improve a specific property, for example, polycarbonates and polyesters [21,22,23], due their high anodic stability. Recently, Gao et al. reported a system polyether (PEO)/poly(ether-acetal) (poly (1,3,6-trioxocane)) with LiTFSI; they showed that these polymers are miscible and, depending on the amount of LiTFSI in the blend, it is possible to obtain immiscible or miscible blends when a critical value is exceeded [24]. When obtaining completely miscible blends, the charge transport is favored.

Previously in our research group it was found that LiTFSI acts as a diluting agent for polyethers [25]. This is of course good for the improvement of the ionic conductivity, but it can compromise the mechanical properties of PEO due to the loss of crystallinity and self-standing character. In this article, a ternary PEO/PHD/LiTFSI system is studied, since both polymers present ionic conductivity when LiTFSI is added [25]. The goal of this article is to investigate the polyether blending and the ionic conductivity and its effect on the crystallinity of the individual homopolymers.

## 2. Materials and Methods

1,6-hexanediol (99%) was purchased from Sigma-Aldrich (Madrid, Spain) and was dried in toluene before using it. Methanesulfonic acid (MSA, 99%), 1,5,7-triazabicyclo [4.4.0] dec-5-ene (TBD, 98%), chloroform (CDCl_3_) and the rest of the solvents used in this work were supplied by Sigma-Aldrich and used as received. Poly(ethylene oxide) (PEO, *M_v_* 100 kg mol^−1^, powder) was purchased from Sigma-Aldrich. Finally, the lithium bis(trifluoromethanesulfonyl)imide (LiTFSI) (99.9%) salt was supplied by Solvionic (Toulouse, France).

### 2.1. Synthesis of PHD: Bulk Self-Condensation of 1,6-Hexanediol

In a 250 mL round bottom flask 0.61 g of MSA and 0.25 g of TBD were weighed and heated at 90 °C for 30 min under vigorous stirring. Once the protic ionic salt was prepared, 20 g of 1,6-hexanediol were added to the flask and it was heated up to 130 °C for 24 h under vacuum. The temperature was increased to 150 °C for the next 24 h and then to 180 °C for the last 24 h. After 72 h the reaction was stopped by cooling it down at room temperature. For the purification, the material was dissolved in chloroform and precipitated in cold methanol. The resulting polymer was filtered and dried it the oven under vacuum at 40 °C, overnight. The ^1^H-NMR spectrum of the obtained polymer and the calculation of the molecular weight are presented in the Supplementary Information (S. I.), Figures S1 and S2.

### 2.2. Blends Preparation

**Scheme 1:**
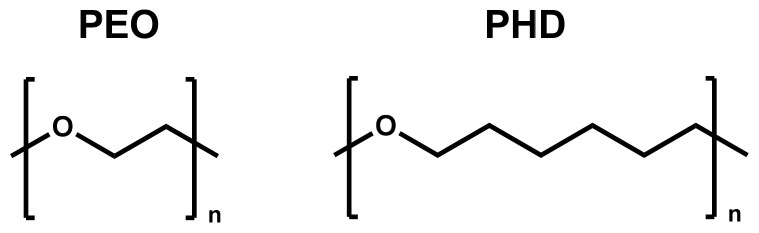
Chemical structure of the aliphatic polyethers used in this work.

Blends between PEO and PHD were prepared by a simple solvent evaporation method (Scheme 1). Even when both polymers are soluble in chloroform, PEO (that tends to aggregate) is slightly less soluble than PHD, so it is added little by little under stirring. A series of 0.5 g samples was prepared with different weight percent of PEO/PHD: 80/20 (0.4 g/0.1 g, 16 mL of CHCl_3_), 60/40 (0.3 g/0.2 g, 14 mL of CHCl_3_), 50/50 (0.25 g/0.25 g, 12 mL of CHCl_3_), 40/60 (0.2 g/0.3 g, 12 mL of CHCl_3_) and 20/80 (0.1 g/0.4 g, 10 mL of CHCl_3_).

The preparation of PEO/PHD blends with LiTFSI salt required some extra work. LiTFSI is indeed soluble in acetone or acetonitrile, but not in chloroform. On the other hand, the opposite is true for PHD, while PEO is soluble both in chloroform and acetonitrile (more easily in the latter). So, the blends with salt were dissolved in an acetonitrile/chloroform mixture, with a vol/vol% roughly equal to the one of the PEO/PHD in the blend. The PHD-rich blends with salt could be successfully dissolved also in a chloroform/acetone (90/10 vol%) mixture. It is worth specifying that the wt% of LiTFSI is referred to the final total weight of the sample (Li salt included).

### 2.3. Characterization

A differential scanning calorimeter (Perkin Elmer DSC 8000, Madrid, Spain) equipped with an Intracooler II was employed to investigate the thermal behavior of the samples. Indium and tin standards were used to calibrate the equipment. For the non-isothermal scans, samples were first heated with a scan rate of 20 °C min^−1^, from 25 to 100 °C, and kept for 3 min at 100 °C to erase thermal history. Then, they were cooled down at 20 °C min^−1^ to −70 °C and subsequently heated at 20 °C min^−1^ at 100 °C. Samples between 5 and 10 mg were used (with higher weights for lower cooling rates), placing them in sealed aluminum pans. Regarding the isothermal DSC experiments, the minimum crystallization temperature (*T_c, min_*) was first determined for each sample, following the procedure recommended by Lorenzo et al. [26]. This temperature represents the minimum one that avoids the crystallization during the cooling step. So, once the starting $T_{c,min}$ is fixed, the isothermal $T_{c}$ range is defined by the other 10 temperatures in the ($T_{c,min}$ + 5 °C) range, with 0.5 °C steps. Therefore, the samples were subjected to: heating from 25 to 100 °C at 20 °C min^−1^; isothermal holding at 100 °C for 3 min; cooling down to the selected $T_{c}$ at 60 °C min^−1^; isothermal holding at that $T_{c}$ until the crystallization process is saturated; and heating from the selected $T_{c}$ to 100 °C at 20 °C min^−1^, in order to register the melting behavior after the isothermal measurement.

X-ray powder diffraction patterns were collected by using a Philips X’pert PRO automatic diffractometer (Madrid, Spain) operating at 40 kV and 40 mA, in theta-theta configuration, secondary monochromator with Cu-Kα radiation (λ = 1.5418 Å) and a PIXcel solid state detector (active length in 2θ 3.347°). Data were collected from 5 to 70° 2θ (step size = 0.026 and time per step = 60 s) at room temperature. A 1° fixed soller slit and divergence slit giving a constant volume of sample illumination were used. The blends were heat treated prior to X-ray measurement, all samples were heated to 100 °C at 20 °C min^−1^ and cooled to room temperature also at 20 °C min^−1^, so that the conditions were the same as in the DSC.

Spherulites nucleation and growth were observed through a polarized light optical microscope (Olympus BX51, Madrid, Spain), equipped with an Olympus SC50 digital camera (Madrid, Spain), with a Linkam-15 TP-91 hot stage (Epsom, England) and coupled to a liquid nitrogen cooling system. Films of thickness roughly equal to 100 µm were prepared by melting the samples between two glass slides. The experimental conditions were very similar to those employed during the DSC isothermal measurements. The samples were heated until 30 °C above their melting point in order to erase their thermal history, and then rapidly cooled down from the melt to the selected isothermal crystallization temperature, $T_{c}$, at 50 °C min^−1^. Finally, the samples were kept at the $T_{c}$ for the time needed to let the spherulites appear and to measure the spherulitic growth rate. This procedure was repeated at 10 different *T_c_*.

Ionic conductivities were measured by electrochemical impedance spectroscopy (EIS) in an Autolab 302N potentiostat galvanostat (Metrohm AG, Herisau, Switzerland), with the temperature controlled by a Microcell HC station. The samples were closed between two stainless steel electrodes (surface area = 0.5 cm^2^). The plots were obtained applying a 10 mV perturbation to open circuit potential in the frequency range of 100 kHz to 1 Hz.

## 3. Results and Discussion

### 3.1. Non-Isothermal DSC of PEO/PHD Blends

PEO/PHD blends were studied in the whole composition range to explore their miscibility and crystallization behavior. One of the first clues that indicate miscibility between two polymers is the appearance of a single glass transition temperature (*T_g_*). However, in this case, both polymers show low *T_g_* (e.g., PEO ≈ −60 °C [27]) and are highly crystalline. For this reason, determining their *T_g_* by DSC is difficult, as the change in specific heat is too small. Nevertheless, the DSC can provide valuable information via the crystallization and melting of the samples.

**Figure 1:**
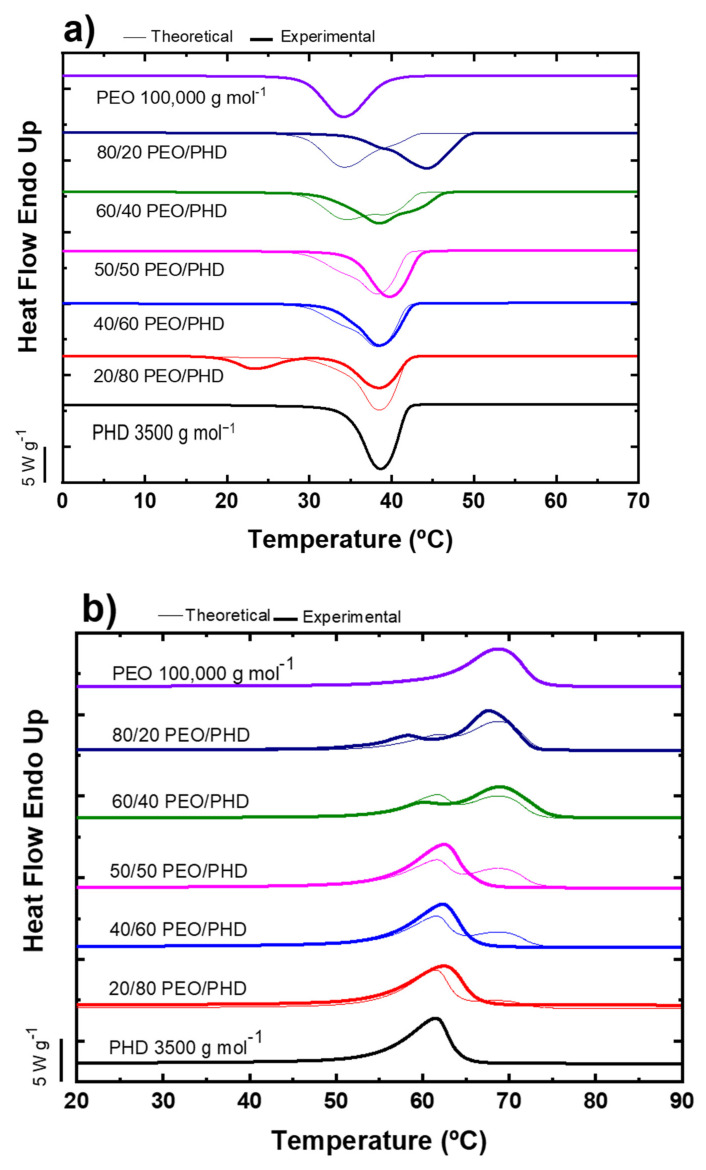
PEO/PHD blends: (**a**) DSC cooling scans at 20 °C min^−1^ and (**b**) DSC second heating scans at 20 °C min^−1^.

The non-isothermal DSC runs were carried out at 20 °C min^−1^. In Figure 1, the experimental curves have been superimposed to scans that were denoted “theoretical”. They were calculated from the experimental DSC scans of the neat homopolymers, they were multiplied by their weight fraction in the blends and then added. In this way, these theoretical curves give an idea of how the DSC traces should appear when there is no interaction whatsoever between the blend components.

The presence of a double peak in Figure 1 indicates that phase separation takes place, while the presence of a single peak in the middle of the neat polymers could in principle indicate a single crystalline phase. Focusing on the neat components, the *T_m_* of PEO is higher than that of the PHD, while the opposite situation is observed in their crystallization temperatures. This is a result of different heterogeneity contents, as this parameter can influence the *T_c_*. On the other hand, *T_m_* is proportional to the lamellar thickness of the crystalline phase (and chemical structure) [28] that is formed, while *T_c_* depends on chemical structure and nucleation density.

**Figure 2:**
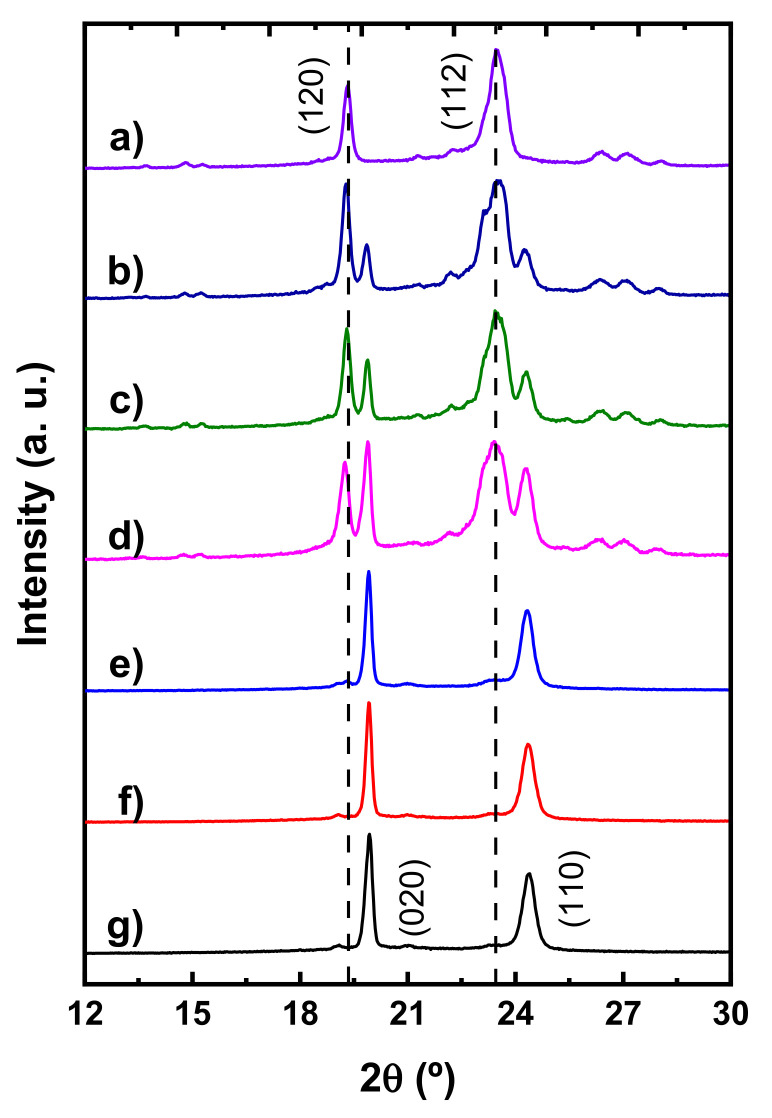
WAXS diffractograms of PEO/PHD blends. (**a**) Neat PEO, (**b**) 80/20 PEO/PHD, (**c**) 60/40 PEO/PHD, (**d**) 50/50 PEO/PHD, (**e**) 40/60 PEO/PHD, (**f**) 20/80 PEO/PHD, (**g**) Neat PHD.

Figure 1a shows the DSC cooling scans from the melt and Figure 1b the subsequent second heating scans. WAXS experiments for the same samples after cooling from the melt at 20 °C min^−1^ can be observed in Figure 2 and will be discussed below. According to Figure 2, in the PEO/PHD blends that are rich in PHD, i.e., 20/80 and 40/60, the only component capable of crystallization is the PHD. This is interesting as it demonstrates that when the blends contain a majority of PHD the crystallization of the PEO phase is hindered and this can be interpreted as a sign of blend miscibility. The 20/80 PEO/PHD blend exhibits two crystallization peaks during cooling from the melt that according to WAXS are both due to PHD crystals. This is an uncommon behavior as fractionated crystallization is normally associated with the minor component in blends [29]. Therefore, this peculiar behavior merits future studies that are outside the scope of the present contribution.

The blends with PEO contents of 50 wt% and higher are all double crystalline blends as demonstrated by WAXS in Figure 2. This means that two crystalline phases are formed and according to Figure 2, the crystalline structure does not change with blend composition (there are no changes in the WAXS reflections). This means that once crystallization starts, phase separation is triggered and within each crystalline phase, the second blend component is completely excluded from the crystals of the component that is crystallizing.

There is a clear nucleating effect of PHD on the PEO phase, as the crystallization peak for PEO shifts to higher temperatures. This nucleation effect is responsible for the overlap of both crystallization peaks for the 50/50 PEO/PHD into a single peak. The melting of this 50/50 blend occurs displaying a single endothermic peak at temperatures slightly higher than the melting peak of neat PHD, but we know by WAXS (Figure 2) that both phases crystallize separately (they do not share the same crystal lattice). Hence, the single melting peak in Figure 1b corresponding to the 50/50 PEO/PHD is due to a coincident melting process of both crystalline phases.

For the 80/20 PEO/PHD blend, the largest crystallization peak in Figure 1a (which should correspond to the PEO phase) is clearly shifted towards higher temperatures. This may suggest that the PHD minor phase acts as a nucleating agent for the main phase (PEO). Since heterogeneous nucleation takes place on a solid phase, this could be due to a transfer of impurities (e.g., polymerization catalyst) present in the PHD phase to the PEO phase. This has already been demonstrated for other blends (e.g., iPP/PS blends [30]). The fact that PHD (even in the molten state) acts as a nucleating agent for PEO is unusual, but a similar behavior has already been reported for other systems in which an amorphous polymer acts as a nucleating agent for a semi-crystalline polymer, for example, atactic polystyrene for polypropylene [31], or poly(vinyl butyral) for poly(butylene succinate) [32].

Both PEO rich blends, i.e., 80/20 and 60/40 PEO/PHD, exhibit clear double endothermic peaks in Figure 1b corresponding to the melting of the PHD crystalline phase and the PEO crystalline phase. The differences between the theoretical and experimental blends indicate that the blends are at least partially miscible with most probably an asymmetrical phase diagram, whose precise determination is outside the scope of the present work. Clearly, the miscibility is higher for the blends with larger contents of PHD and limited for blends with higher PEO contents.

### 3.2. Wide Angle X-ray Scattering of PEO/PHD Blends

The WAXS study was carried out for the blends to determine if there are any changes in the crystal structure of the neat polymers when mixed. In Figure 2, the diffraction patterns of the polymers and blends at 25 °C are presented. The samples were first heated to the melt and then cooled at 20 °C min^−1^ to reproduce similar conditions to the samples in Figure 1a.

The reflections of neat PEO are observed at 19.4° and 23.45°, which are assigned to the (120) and (112) planes, respectively [33,34,35], and the reflections of neat PHD are presented at 19.85° and 24.29°, and are assigned to the (020) and (110) planes, respectively [36,37]. The blends 80/20, 60/40, 50/50 PEO/PHD show the peaks of both crystalline structures without any changes in the 2θ angles, which indicates the two phases crystallize separately and without any modification of the crystalline structure of the neat blend components. On the other hand, in the 40/60 and 20/80 PEO/PHD blends, only the characteristic reflections for PHD are presented, which suggests that the presence of PHD prevents the crystallization of PEO in the blends, possibly because the blends exhibit miscibility in the melt state.

### 3.3. Morphology and Crystal Growth Rate

**Figure 3:**
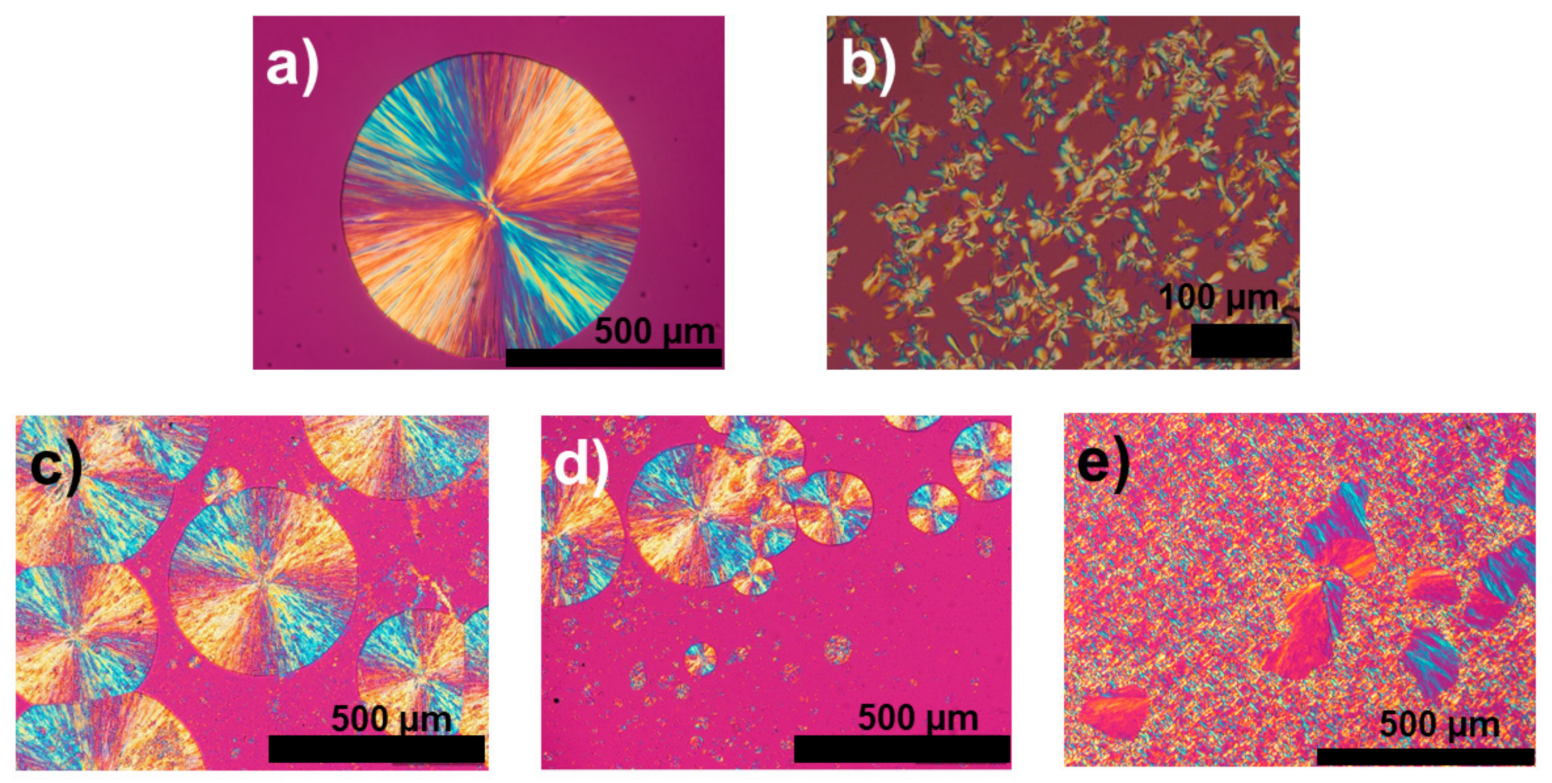
PLOM micrographs obtained during isothermal crystallization at 40 °C. (**a**) PEO, (**b**) PHD, (**c**) 80/20 PEO/PHD blend, (**d**) 50/50 PEO/PHD blend, and (**e**) 20/80 PEO/PHD.

The morphology and spherulitic growth rate were analyzed by polarized light optical microscopy (PLOM). With this technique it is possible to measure the isothermal growth of spherulites from the melt in samples that do not have a very large nucleation density. Figure 3a,b show that PEO forms large spherulites, while PHD forms small axialites with high nucleation density. The superstructures growth rate (e.g., spherulites or axialites) depends on a competition between secondary nucleation and diffusion [38,39].

Figure 3 shows the PLOM images for the neat homopolymers and three different compositions, obtained at 40 °C. Neat PEO presents as expected large negative spherulites (Figure 3a), while PHD crystallizes in small axialites (Figure 3b). The composition 80/20 PEO/PHD (Figure 3c), shows a larger number of the PEO phase spherulites as compared with neat PEO, confirming the nucleation effect of PHD addition reported in Figure 1a. The presence of very small PHD phase axialites can also be observed. Figure 3d presents the morphology of the 50/50 PEO/PHD blend, where PEO phase larger spherulites can be clearly seen. Some small PHD phase axialites are also observed, indicating that both phases can crystallize. These results for the 80/20 and 50/50 PEO/PHD blends show that both components are able to crystallize under isothermal conditions and are consistent with DSC and WAXS results taken after non-isothermal crystallization.

Finally, for the 20/80 PEO/PHD blend, Figure 3e shows that although most of the optical view field is filled with PHD phase axialites, there are some large birefringent structures that are probably constituted by the PEO phase. This is an unexpected observation, as in Figure 1 and Figure 2, this sample under non-isothermal conditions has a different behavior, as only the PHD phase is able to crystallize. Apparently, under isothermal conditions, and given enough time, both phases can eventually crystallize. Nevertheless, as the majority of the sample crystallizes with very small PHD phase axialites, growth rate measurements proved to be impossible.

The spherulitic growth rate measurements were possible only for neat PEO and the PEO-rich compositions (80/20 and 50/50 PEO/PHD) but not for the rest of the samples, as their nucleation density was too high.

**Figure 4:**
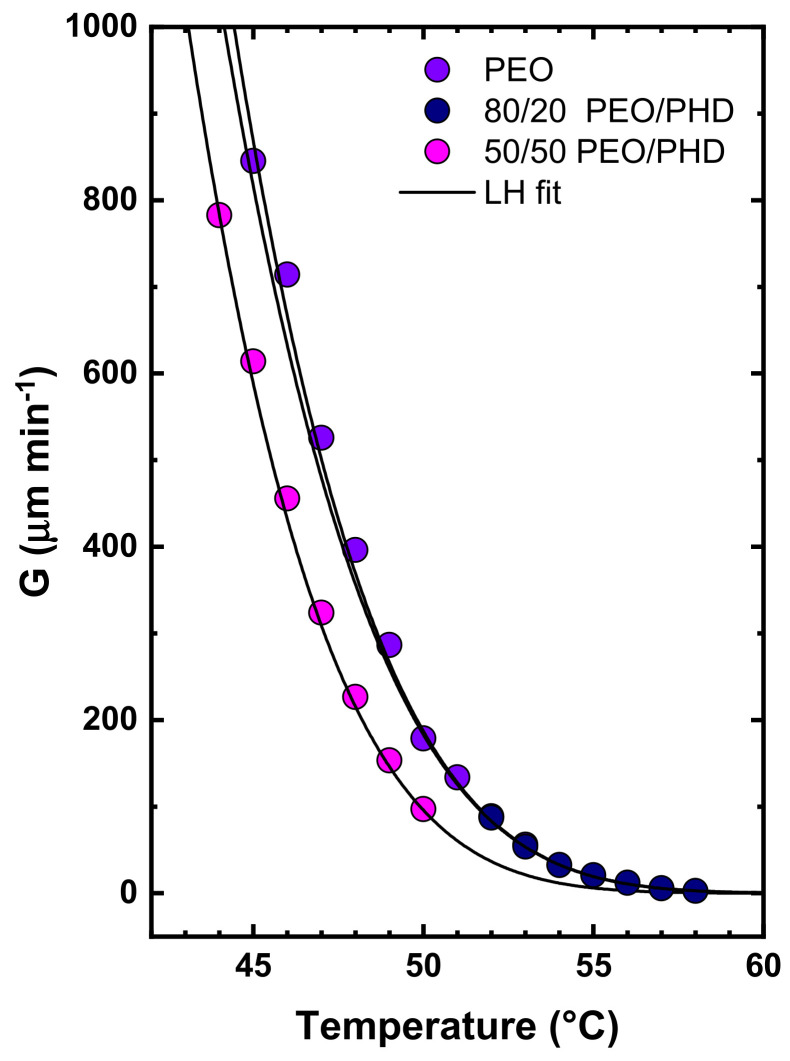
Spherulitic growth rate (G) as a function of isothermal crystallization temperature for neat PEO and for the PEO component of the 80/20 and 50/50 PEO/PHD blends.

Figure 4 shows the spherulitic growth rate as a function of temperature and the solid lines are fits to the Lauritzen and Hoffman theory [40,41], see also the S.I. The spherulitic growth rate of the 80/20 PEO/PHD blend matches perfectly with that of neat PEO, a result that indicates immiscibility between the two components for this composition. This result is in line with the DSC melting curves of Figure 1b that show a similar behavior between the experimental and the theoretical immiscible blend DSC heating scans.

On the other hand, Figure 4 also shows the spherulitic growth rate versus temperature for the 50/50 PEO/PHD blend, where a clear decrease in the PEO phase growth rate is observed with respect to neat PEO. This result can be taken as an evidence of miscibility between PEO and PHD components, because if they were immiscible, no change in the PEO spherulites growth rate would be expected.

### 3.4. Non-Isothermal DSC of PEO/PHD Blends with LiTFSI

**Figure 5:**
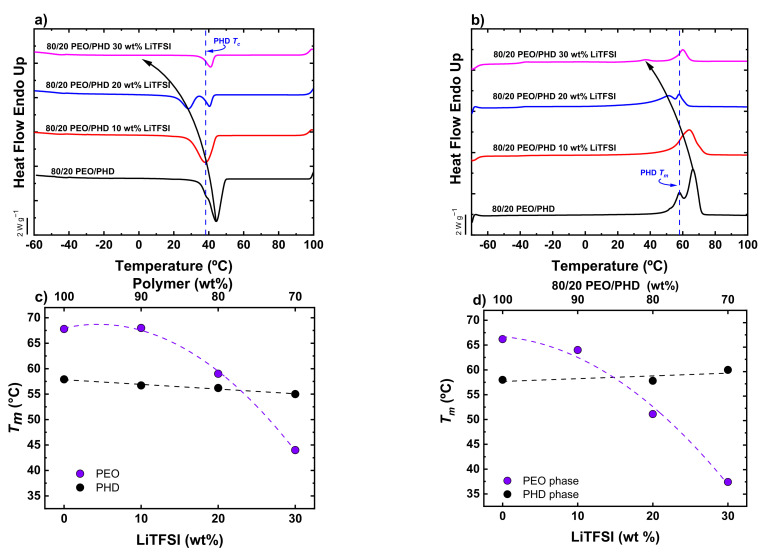
DSC for the PEO/PHD blends: (**a**) during cooling from the melt, (**b**) subsequent melting scans, (**c**) change in *T_m_* for the PEO and PHD homopolymers as a function of LiTFSI concentration, (**d**) change in *T_m_* for the PEO and PHD phases within the 80/20 PEO/PHD as a function of LiTFSI concentration.

LiTFSI salt was added to the 80/20 PEO/PHD blend to evaluate the effect of this salt on the crystallization. In previous work, we demonstrated that LiTFSI acts as a diluent agent that depresses the *T_m_* in these polyethers [25] (Figures S3 and S4). Figure 5a shows cooling scans from the melt for the 80/20 PEO/PHD blend with different concentrations of LiTFSI (10, 20 and 30 wt% LiTFSI).

In the 80/20 PEO/PHD blend without salt, one main crystallization peak is observed, which results from the crystallization of the PEO phase. It is also possible to observe a low temperature shoulder that corresponds to the crystallization of the PHD phase. Upon salt addition, the *T_c_* of the PEO phase decreases as a function of the LiTFSI concentration (see the arrow that guides the eye in Figure 5a), whereas the *T_c_* of the PHD phase remains constant (approx. at 40 °C, see the dashed vertical line in Figure 5a). The same effect is observed in the fusion behaviour shown during the second DSC heating scans (see Figure 5b), where the *T_m_* values of the PEO phase decreases (see the arrow that guides the eye in Figure 5b) while the *T_m_* value of the PHD phase remains constant at 58 °C (see the vertical dashed line in Figure 5b). The above described trends of *T_m_* as a function of salt content can be clearly observed in Figure 5c,d, respectively.

The results shown in Figure 5 indicate that the lithium salt prefers to dissolve in the PEO phase rather than in the PHD phase within the 80/20 PEO/PHD blend. This is also corroborated by the decrease in the enthalpies of crystallization and melting of the PEO phase as the salt concentration increases, while those of the PHD phase do not seem to be altered (although the overlapping of signals make this observation difficult). The behaviour of this blend with lithium salt could be a good solution to the mechanical stability problems that these types of electrolytes present for lithium batteries, as even at high lithium concentrations, the PHD phase remains semi-crystalline, while the PEO/lithium phase becomes fully amorphous at 30% lithium loadings.

### 3.5. Isothermal Crystallization Studies of the PEO/PHD Blends with LiTFSI

The spherulitic growth rate was measured for the 80/20 PEO/PHD blend with LiTFSI, but it was only possible to measure the blend with 10 wt% LiTFSI, because there is very high nucleation density with higher amounts of salt and it is not possible to follow the growth of the very small spherulites that quickly impinged on one another. In the SI some PLOM images are shown for PEO and PHD with 20 wt% LiTFSI samples.

**Figure 6:**
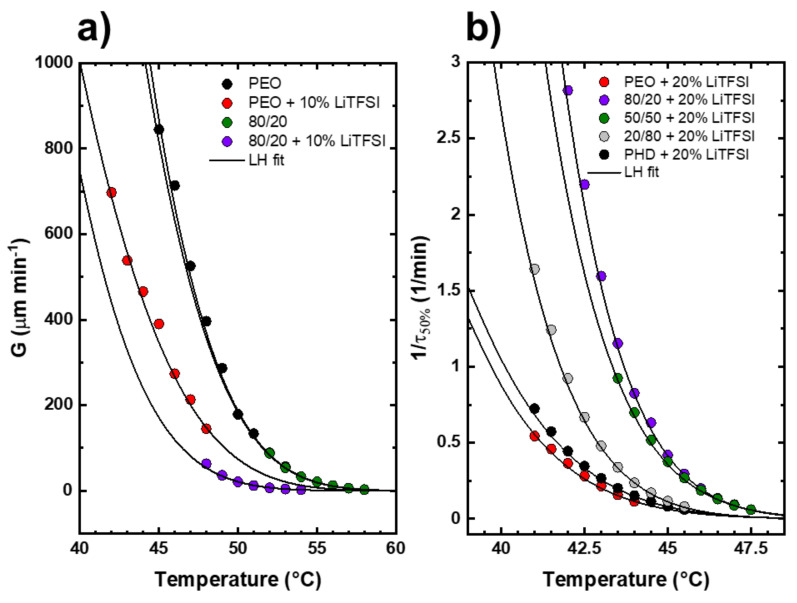
(**a**) Spherulitic growth rate of PEO and 80/20 PEO/PHD blends with and without 10% LiTFSI, (**b**) Overall crystallization rate of homopolymers and blends with 20 wt% LiTFSI.

Figure 6a shows the spherulitic growth rate as a function of crystallization temperature and the solid lines represent the Lauritzen–Hoffman equation fit. The general trend is that the spherulitic growth rates of neat PEO and the PEO phase within the 80/20 PEO/PHD blend decrease with LiTFSI addition, although the decrease in much higher in the blend. This is because the salt prefers to dissolve in the PEO phase and, consequently, the LiTFSI concentration in PEO is higher in the blend than in neat PEO.

Isothermal crystallization studies by DSC take into account both nucleation and growth of crystals. Figure 6b shows the inverse of the half-crystallization time (1/τ_50%_), which represents the overall crystallization rate, as a function of crystallization temperature (*T_c_*), for PEO, PHD and PEO/PHD blends with 20 wt% LiTFSI. This salt concentration was selected in order to have a clearer comparison of the effect of the salt in the blends (the effect of salt concentration in the homopolymers is presented in the S.I., Figure S5).

It is observed that the homopolymers with salt exhibit the lowest values of overall crystallization rates. The blends, however, experience a large increase in overall crystallization as the amount of PEO in the blends increases. As indicated in Figure 6a, the spherulitic growth rate decreases with 10% salt addition. We speculate that this decrease should be equal or higher with 20% salt addition. Therefore, the synergistic increase in overall crystallization rate shown in Figure 6b when 20% LiTFSI is added to the blends must be due to a very large nucleation effect that offsets the crystal growth rate retardation. The nucleation effect could be due to the transfer of impurities from the PHD phase to the PEO phase.

### 3.6. Ionic Conductivity

Ionic conductivity is a very important parameter for the use of SPEs, ionic conductivity in polymers takes place in the amorphous phase, and the crystalline phase restricts ionic conductivity [24], for this reason, amorphous systems are normally used for this application.

**Figure 7:**
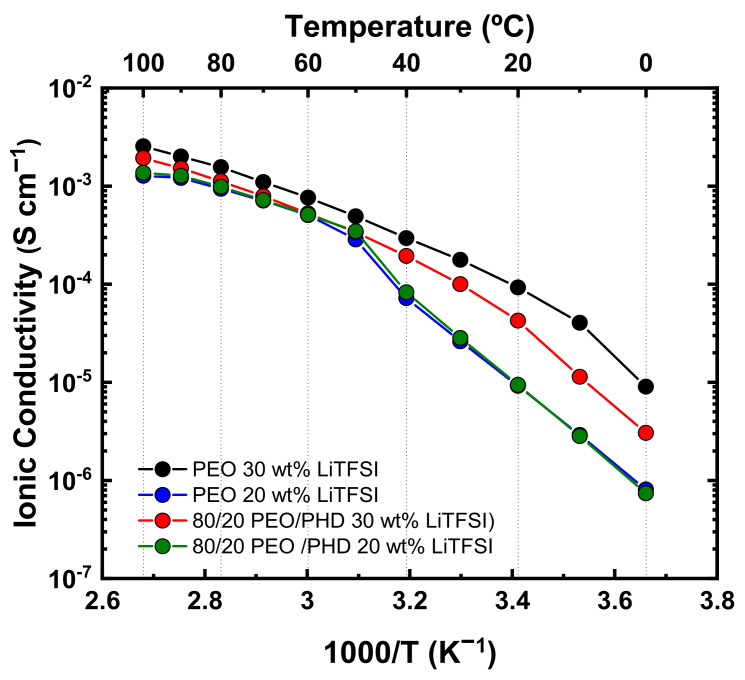
Ionic conductivity as a function of temperature for PEO and 80/20 PEO/PHD blends with the either 20 or 30 wt% LiTFSI, as indicated in the inner legend.

Ionic conductivity was measured by Electrochemical Impedance Spectroscopy (EIS) for the 80/20 PEO/PHD blends with 20 and 30 wt% LiTFSI, and the values were compared to those obtained with those for neat PEO with salt. Figure 7 shows the values of ionic conductivity as a function of temperature for the different blends. PEO presents the highest value of ionic conductivity with 30 wt% LiTFSI, 1.1×10^−3^ S cm^−1^ at 70 °C. It should be recalled that with such a high salt concentration, PEO does not crystallize and remains amorphous over the entire range of temperatures where the measurements were performed. Furthermore, it is important to note that, with 20 wt% LiTFSI, PEO can crystallize (although up to a small degree) and therefore presents a small drop in conductivity values in the temperature range where it crystallizes.

In the PEO/PHD blends, there is a drop in the conductivity values at 40 °C (Figure 7), which is the temperature at which PHD begins to crystallize. These results are consistent to those obtained by DSC (Figure 5). Besides, adding 20 or 30 wt% LiTFSI makes no difference in the ionic conductivity of the polymer blend, perhaps due to a saturation of lithium salt that can be dissolved in the PEO phase [42]. The ionic conductivity values at high temperatures (>60 °C) for the mixtures are very similar, around ~10^−3^ S cm^−1^. At lower temperatures, a difference is observed; for example, at 20 °C the ionic conductivity of the blends PEO/PHD is 9.5×10^−6^ S cm^−1^, while the PEO references present a higher ionic conductivity. These results are similar to those presented by Gao et al. [24], where in a PEO/poly (ether-acetal) blend, the lithium salt prefers to dissolve in PEO, showing conductivities similar to PEO with LiTFSI.

Even though the conductivity values obtained for the 80/20 PEO/PHD blend with 30% LiTFSI are lower than for PEO in the range below 40 °C, we must remember that in the blends, at temperatures below the melting point of PHD, the crystals of PHD will probably reinforce the electrolyte, hence the material will exhibit better mechanical properties.

## 4. Conclusions

In the present work, blends of PEO and PHD were prepared by a solvent evaporation method. Taking into account the results obtained by DSC, PLOM and WAXS, it can be concluded that the blends are partially miscible, and the miscibility is a function of blend composition. Blends with higher amounts of PEO have limited miscibility and are double crystalline, while those with higher amounts of PHD exhibit improved miscibility and only the PHD phase can crystallize in them. The PHD phase can nucleate the PEO phase probably by a transference of impurities mechanism.

The addition of a lithium salt (LiTFSI) on the neat polymers and the blends was also studied. The results indicate that the lithium salt preferentially dissolves in the PEO phase and has an insignificant effect on the PHD component, this effect could be one of the keys to improve the mechanical properties of these materials. Even though the salt reduces the spherulite growth rate of the PEO phase within the blends, the overall crystallization rate is enhanced as a result of the strong nucleating effect of the PHD component.

In terms of ionic conductivity, at high temperatures, the conductivity is in the order of ~10^−3^ S cm^−1^, and as the temperature decreases, the crystallization of PHD is observed.

## Supplementary Materials

The following are available online at https://www.mdpi.com/article/10.3390/polym13132097/s1, Figure S1: NMR of PHD, Figure S2: GPC analysis of PHD, Figure S3: DSC of PEO and PHD with different LiTFSI concentrations, Figure S4: PEO/PHD blends with LiTFSI at PLOM, (a) 80/20 with 20 wt% LiTFSI and (b) 50/50 with 20 wt% LiTFSI, Figure S5: Overall crystallization rate by DSC for neat PEO and 80/20 PEO/PHD with different LiTFSI concentration.
