# Supplementary material for: Polyether Single and Double Crystalline Blends and the Effect of Lithium Salt on Their Crystallinity and Ionic Conductivity
Source: Polymers (Basel). 2021 Jun 25;13(13):2097. doi: 10.3390/polym13132097 (PMC8271483; doi:10.3390/polym13132097)
Supplement: Supplementary file 1 [file polymers-13-02097-s001.zip › polymers-1270846-supplementary.pdf]

Supplementary information

Polyether single and double crystalline blends and the effect of lithium salt on their crystallinity and ionic conductivity

By

Jorge L. Olmedo-Martínez[a,1], Michele Pastorio[a,c,1], Elena Gabirondo[a], Alessandra Lorenzetti[c], Haritz Sardon[a], David Mecerreyes[a,b], Alejandro J. Müller[a,b] *

[a] POLYMAT and Department of Polymers and Advanced Materials: Physics, Chemistry and Technology, Faculty of Chemistry, University of the Basque Country UPV/EHU, Paseo Manuel de Lardizabal 3, 20018 Donostia-San Sebastián, Spain

[b] IKERBASQUE, Basque Foundation for Science, 48011 Bilbao, Spain

[c] Departement of Industrial Engineering, University of Padova, via Marzolo, 9, Padova 35131, Italy

[1] These authors contributed equally to this work.

Nuclear Magnetic Resonance (NMR) Spectroscopy.

1H nuclear magnetic resonance (NMR) spectra were recorded in a Bruker Avance DPX 300 at 300.16 MHz of resonance frequency using deuterated chloroform ($CDCl_3$) as solvent at room temperature. Experimental conditions were as follows: 10 mg of sample; 3 s acquisition time; 1 s delay time; 8.5 μs pulse; spectral width 5000 Hz and 32 scans.

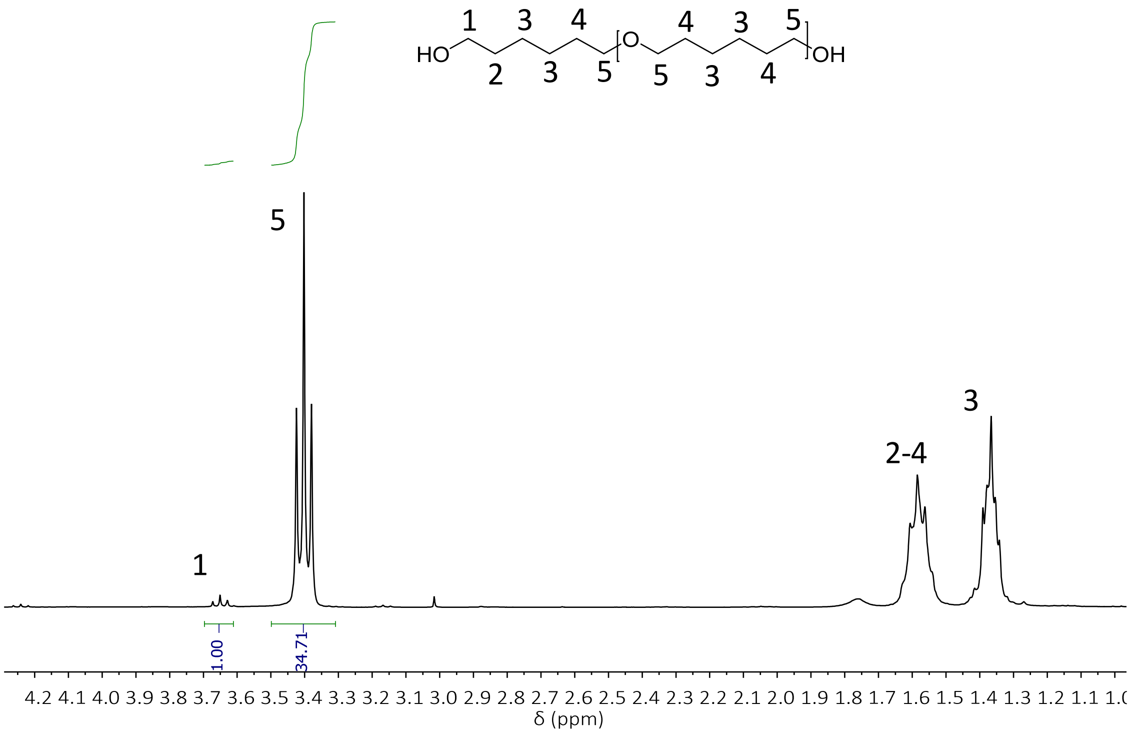

Figure S1. ^{1}H-NMR of PHD.

Calculation of molecular weight by ^{1}H-NMR

The signal of the ester (signal 5) was related with the proton that is next to the alcohol group, in order to know the value of the repeating units (n):

$$n = \frac{Contribution\ of\ signal\ 5}{Contribution\ of\ signal\ 1} = \frac{34.71}{1} = 34.71$$

Afterwards, the repeating unit value is multiplied by the molecular weight of the repeating unit and added the ending group molecular weight (18 g mol^{-1}):

$$Mn = (n * 100.17) + 18$$

$$Mn = (34.71 * 100.17) + 18 = 3494.90\ g\ mol^{-1}$$

The molecular weight of the polymer by ^{1}H-NMR is 3500 g mol^{-1}.

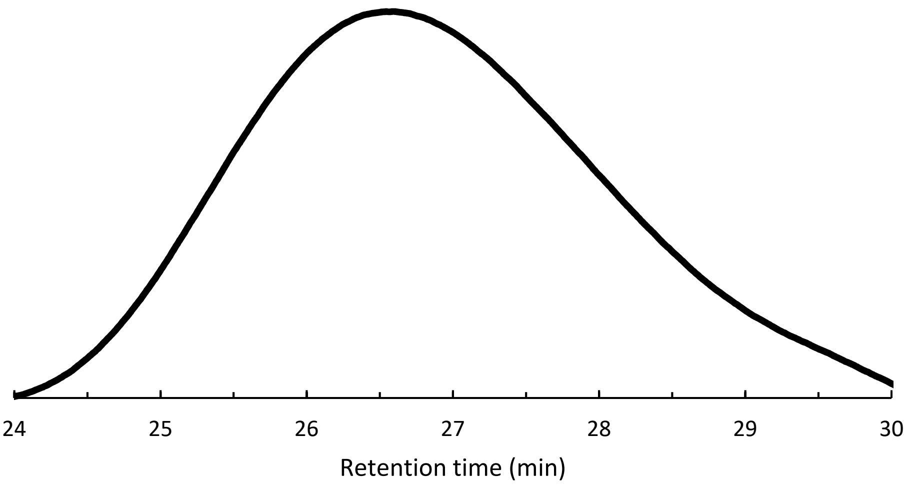

Figure S1. GPC analysis of PHD.

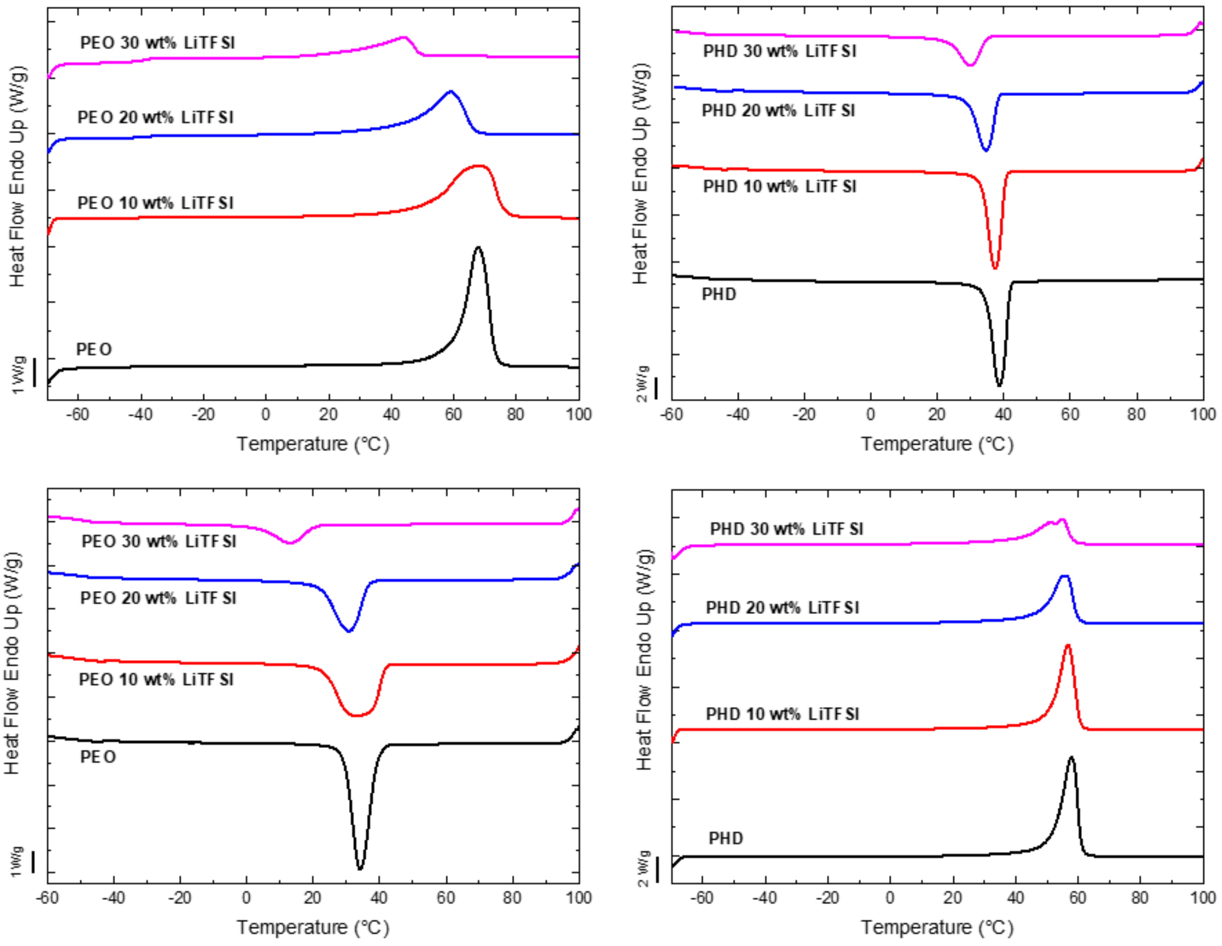

Figure S3. DSC of PEO and PHD with different LiTFSI concentrations.

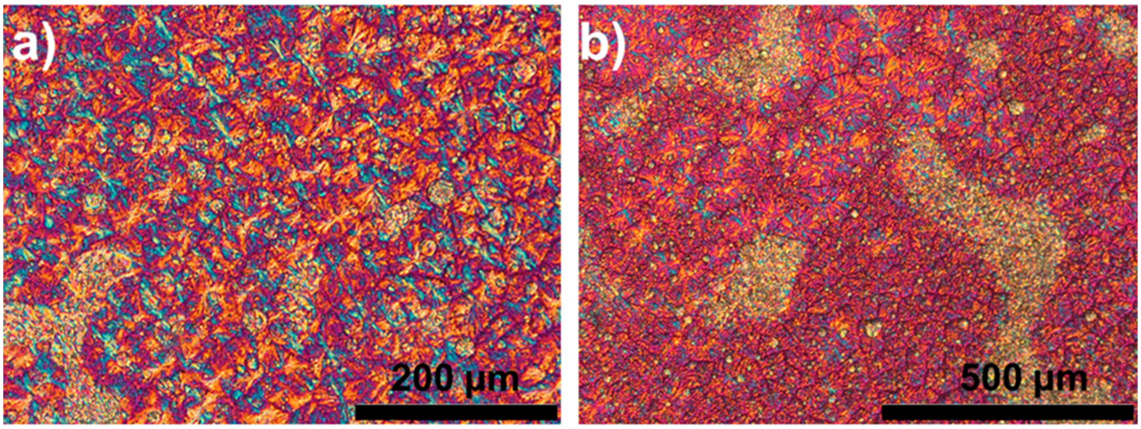

Figure S4. PEO/PHD blends with LiTFSI at PLOM, a) 80/20 with 20 wt% LiTFSI and b) 50/50 with 20 wt% LiTFSI.

Spherulitic growth rate

In all cases, the experimental values were fitted with the Lauritzen-Hoffman equation, in which spherulite growth can be expressed as follows:

$$G = G_0(\Delta T)\exp\left(\frac{-U^*}{R(T_c - T_\infty)}\right)\exp\left(\frac{-K_g}{T_c \Delta T f}\right)$$

Where, G_0 is a pre-exponential factor, ΔT is the degree of supercooling (T_m^0 - T_c) with T_m^0 being the equilibrium melting temperature and T_c the crystallization temperature. U^* is the activation energy needed for polymer diffusion, R is the universal gas constant, T is the temperature below which all motions freeze (taken as T_g - 30 K), and f is a temperature correction factor accounting for the change of melting enthalpy with temperature, which is given by the expression $2T_c$ / (T_m^0 + T_c,) and K_g is a constant.

Isothermal crystallization

In all cases, the experimental values were fitted with the Lauritzen-Hoffman equation, the inverse of the experimental half-crystallization time ($1/\tau_{50\%}$) can be expressed as a function of ΔT as follows:

$$1/\tau_{50\%}(T) = 1/\tau_0 \ \exp\left(\frac{-U^*}{R(T_c - T_\infty)}\right)\exp\left(\frac{-K_g^\tau}{T_c \Delta T f}\right)$$

Where, $1/\tau_0$ is a pre-exponential factor. U* is the activation energy for the transport of the chains to the growing front (U* = 1500 cal mol^{-1}), R is the gas constant, T_c is the

isothermal crystallization temperature, T_∞ is the temperature at which chain mobility ceases (taken as T_g-30 K), ΔT is the supercooling defined as the difference between equilibrium melting point (T_m^0) and the isothermal crystallization temperature; f is a temperature correction factor and K_g^τ is a constant which is proportional to the energy barrier for both nucleation and growth.

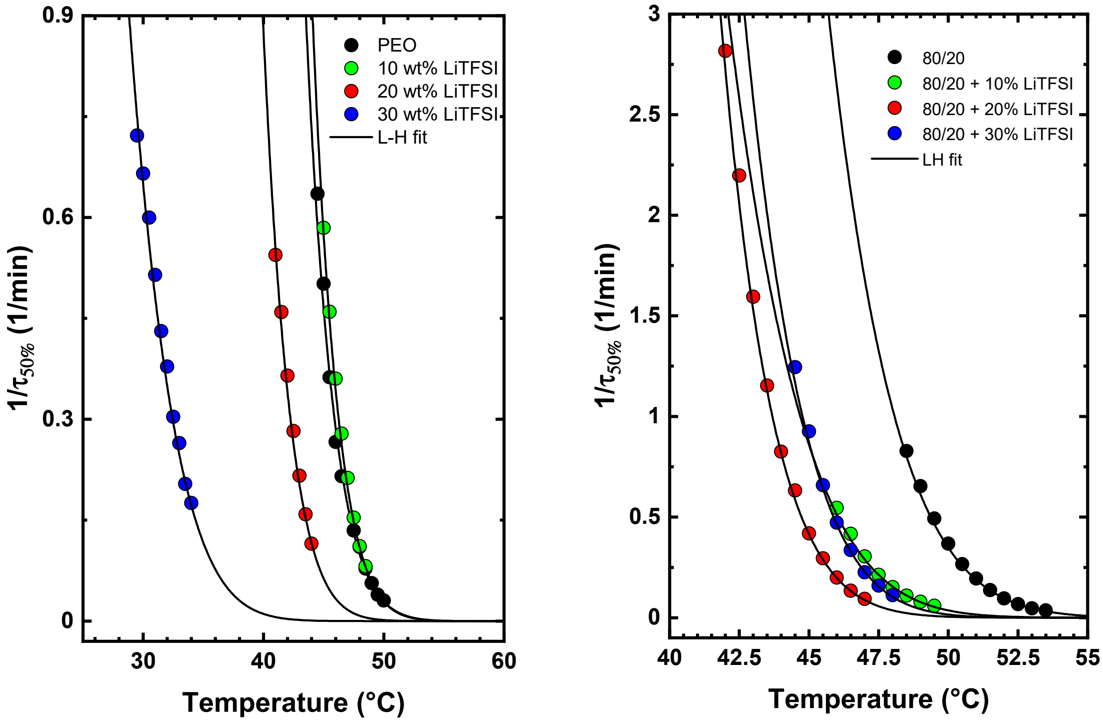

Figure S5. Overall crystallization rate by DSC for neat PEO and 80/20 PEO/PHD with different LiTFSI concentration.
